# Polarization-Gradient Engineering of InGaN Nanorods for Hybrid Mechanical–Optical Energy Harvesting

**DOI:** 10.1021/acsnano.6c09038

**Published:** 2026-08-31

**Authors:** Sheng-Shong Wong, Zhen-You Lin, Ya-Chi Chiu, Ming-Hao Lee, Ming-Wen Chu, Chia-Hao Chen, Chung-Lin Wu

**Affiliations:** † Department of Physics, 34912 National Cheng Kung University, Tainan 70101, Taiwan; ‡ 57815 National Synchrotron Radiation Research Center (NSRRC), Hsinchu 30076, Taiwan; § Center for Quantum Frontiers of Research and Technology (QFort), National Cheng Kung University, Tainan 70101, Taiwan; ∥ The Key Consortium of Electron Microscopy, 33561 National Taiwan University, Taipei 10617, Taiwan; ⊥ Center for Condensed Matter Sciences and Center of Atomic Initiative for New Materials, National Taiwan University, Taipei 10617, Taiwan

**Keywords:** InGaN nanorods, polarization gradient, piezoelectric nanogenerator, piezo-phototronics, band bending, AR-HAXPES

## Abstract

Integrating mechanical and optical energy harvesting within a single piezoelectric semiconductor platform remains challenging because photoexcited carriers readily screen the piezoelectric potential and suppress nanogenerator output. Here, we realize highly crystalline, polarity-defined N-polar InGaN/GaN nanorods with opposite axial In gradients and correspondingly opposite built-in electrostatic profiles. Depth-sensitive, angle-resolved hard X-ray photoelectron spectroscopy (AR-HAXPES) reveals opposite equilibrium band-bending profiles—surface carrier accumulation in In-increasing nanorods but depletion in In-decreasing ones—showing that polarization grading redistributes as-grown mobile carriers and that only the In-increasing configuration establishes the internal electrostatics needed to suppress carrier screening and preserve the piezoelectric potential during nanogenerator operation. In vertically integrated nanogenerators (VINGs), this asymmetry produces a much larger output from the In-increasing device than from the In-decreasing one, together with more favorable transient behavior and energy-storage performance, and is further amplified under illumination, which enhances the output of the In-increasing device while suppressing that of the In-decreasing one, consistent with opposite photoscreening dynamics set by the grading-defined built-in fields. These results demonstrate hybrid mechanical–optical energy harvesting in direct-band gap piezoelectric semiconductors such as III-nitrides, where light and strain act cooperatively rather than competitively.

## Graphical abstract

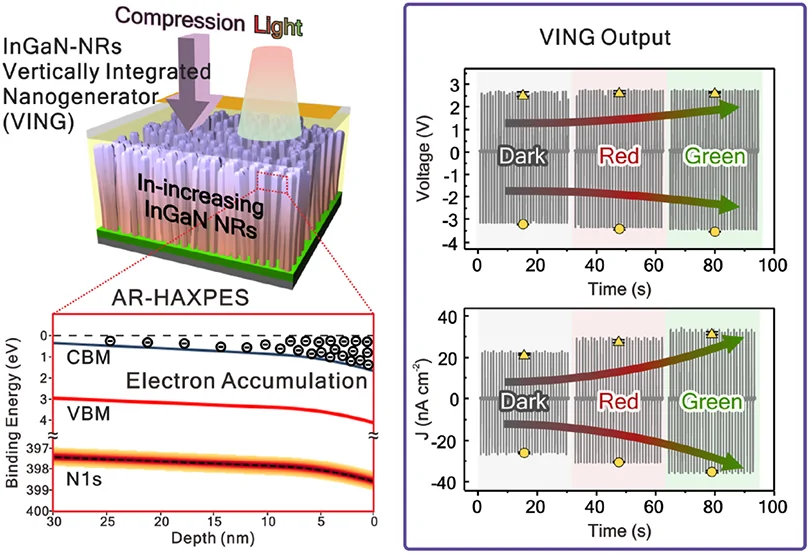

## Introduction

A central challenge in piezoelectric semiconductor energy harvesting is that the mobile carriers required for electrical functionality also screen the strain-induced piezoelectric potential needed for energy conversion.^1–4^ Under illumination, this limitation becomes even more pronounced because photoexcited carriers further weaken the internal fields required for device operation, making the integration of mechanical and optical energy harvesting within a single semiconductor architecture inherently difficult,^5–8^ despite the promise of such systems for self-powered electronics,^9,10^ distributed sensing,^11,12^ and autonomous microsystems.^13–15^ Existing strategies have therefore relied on surface, interface, or junction engineering, including passivation,^7,16^ asymmetric Schottky/MSM geometries,^17,18^ and p–n heterojunctions that enhance carrier separation and suppress recombination.^19,20^ Although effective, these approaches generally require additional processing or structurally elaborate device designs rather than directly engineering the internal electrostatics of the piezoelectric semiconductor itself. A more intrinsic strategy is polarization-gradient engineering in piezoelectric semiconductors, which establishes the internal electrostatics in advance to redistribute mobile carriers rather than allowing them to screen the piezoelectric potential during operation. III-nitride semiconductors are especially attractive for hybrid mechanical–optical energy harvesting because they combine strong polarization effects^21–23^ with direct band gaps and broad alloy tunability.^24–26^ In particular, InGaN is well suited for this purpose because its alloy composition-dependent band gap can cover the visible spectral range, making it an attractive platform for coupling piezoelectric functionality with solar-light harvesting. Because the spontaneous and piezoelectric polarizations vary with alloy composition between GaN and InN, composition grading along the polar *c*-axis of InGaN produces a spatial gradient in polarization, thereby inducing bound charges distributed within the graded region and providing an internal electrostatic control parameter with doping-like functionality without relying solely on impurity incorporation.^27–29^ This concept becomes particularly powerful in InGaN nanorod architectures, where free sidewalls promote elastic strain relaxation, suppress mismatch-induced defects and parasitic strain fields,^30,31^ and enable more independent control of composition, polarity, strain state, and internal field distribution than in planar heterostructures,^30,32–34^ while the mechanically compliant geometry is also inherently favorable for hybrid nanogenerator operation.^35,36^ Yet whether the internal electrostatics imposed by grading direction can be measured directly—and shown to govern device behavior—has remained unclear. Here, we address this issue in polarity-defined, axially graded N-polar InGaN/GaN nanorods using angle-resolved hard X-ray photoelectron spectroscopy (AR-HAXPES), which directly resolves the unloaded electrostatic band-bending profile. The measurements show that reversing the grading direction reverses the unloaded band bending, thereby establishing the electrostatic origin of the contrasting nanogenerator responses. Conceptually, this work shows that alloy engineering in III-nitride nanostructures extends beyond its conventional role of band gap tuning for solar-light harvesting and optoelectronic functionality, enabling internal electrostatics to be engineered as an active design parameter for hybrid energy harvesting.

## Results and Discussion

To establish polarization-gradient-defined electrostatics in a geometry suited to mechanical energy harvesting and isolate the role of axial In-composition grading direction, we prepared polarity-defined InGaN/GaN nanorods with either In-increasing or In-decreasing axially graded InGaN regions. The free sidewalls of the nanorod geometry facilitate lateral elastic relaxation and reduce the formation of mismatch-induced extended defects compared with planar heterostructures. The nanorods were grown on n-type Si(111) by plasma-assisted molecular beam epitaxy (PA-MBE) under N-rich conditions, with the growth sequence monitored in situ by reflection high-energy electron diffraction (RHEED), which tracks the transition from the Si(111)-(7 × 7) reconstructed substrate to GaN nanorod nucleation and finally to the spotty diffraction characteristic of vertically aligned graded InGaN/GaN heterostructures (Figure 1a).^37–39^ Plan-view and cross-sectional SEM reveal dense, vertically aligned arrays with closely matched morphology for both grading directions, including diameters of ∼100 nm, areal densities of ∼3 × 10^10^ cm^–2^, and lengths exceeding 800 nm after 8 h, thereby enabling direct comparison of grading-direction effects (Figure 1b,c). Secondary ion mass spectrometry (SIMS) and room-temperature photoluminescence (PL) verify the intended axial In gradients; the SIMS-derived composition profiles are monotonic but not linear. Because 325 nm excitation laser probes only the upper tens of nanometers, PL mainly reflects the near-surface composition, giving visible emission at ∼464 nm for the In-increasing nanorods but near-GaN emission at ∼365 nm for the In-decreasing ones, consistent with the opposite near-surface compositions imposed by the two grading directions. High-resolution transmission electron microscope (HR-TEM) and annular bright-field scanning transmission electron microscope (ABF-STEM) images reveal highly crystalline nanorods free of discernible defects, together with a clear increase in the *c*-axis lattice spacing from ∼5.196 Å in the GaN base to ∼5.306 Å in the upper InGaN region (Figure 1d) of the In-increasing graded InGaN/GaN nanorod. Assuming a fully relaxed lattice, this expansion corresponds to a local In content of ∼24%, in excellent agreement with the SIMS profile. High-magnification STEM further identifies the nanorods as N-polar,^32,33,40,41^ with the weaker-contrast N atomic columns located directly above the stronger-contrast metal columns along the [0001̅] growth direction. This observation is fully consistent with the well-established N-polar growth mode of PA-MBE-grown III-nitride nanorods on Si(111),^1,32,33,42^ thereby establishing the structural and polarity-defined foundation for polarization-gradient electrostatic engineering.

**Figure 1:**
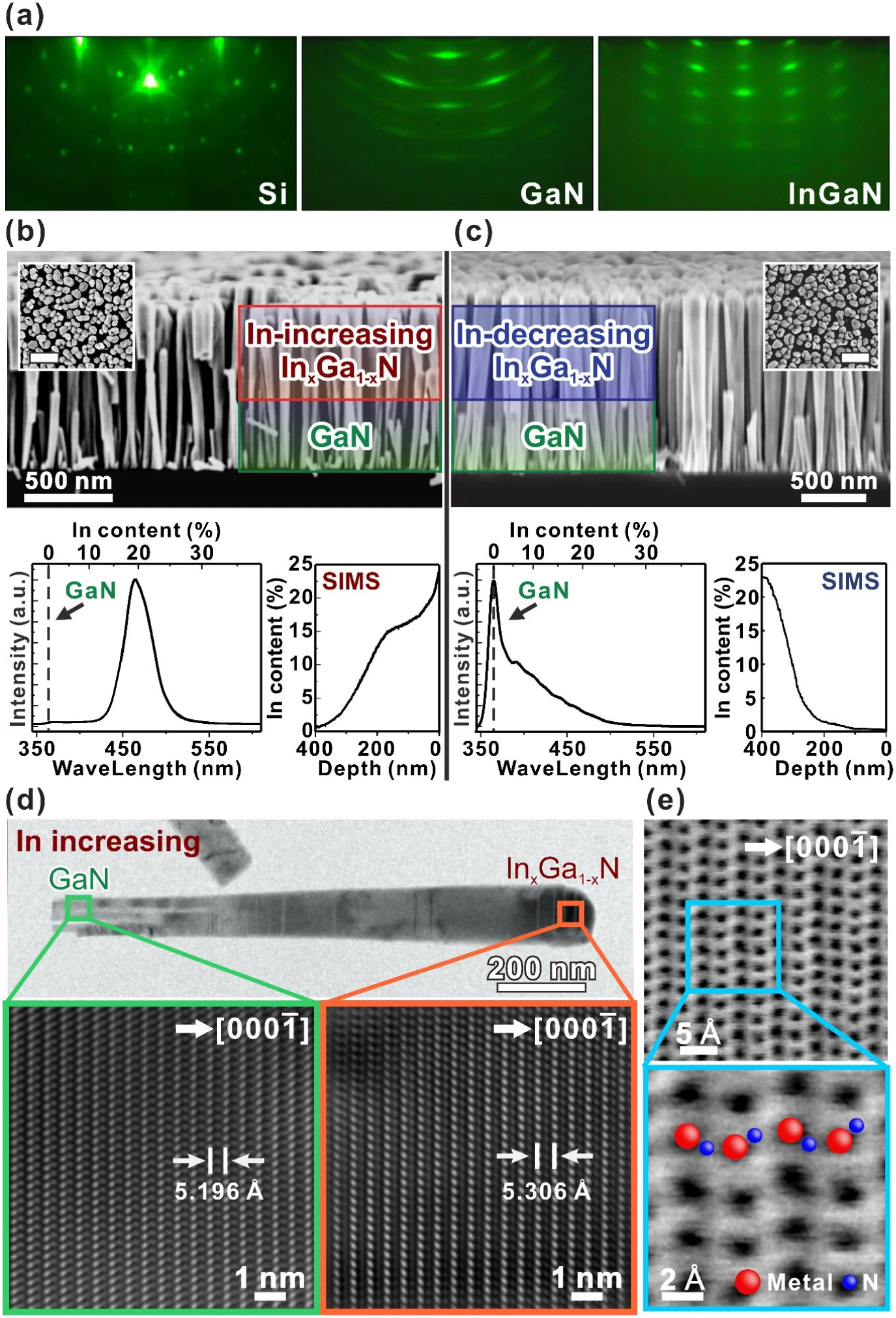
Epitaxial growth of N-polar InGaN/GaN on Si(111). (a) RHEED patterns taken along the InGaN [112̅0] direction, showing the evolution from the initial Si 7 × 7 reconstructed surface (left) to the GaN buffer layer (middle) and finally to the InGaN epilayer (right). (b, c) Cross-sectional SEM images of the In-increasing (b) and In-decreasing (c) InGaN nanorods, with the corresponding top-view SEM images shown in the insets. The scale bars in the inset images correspond to 500 nm. The lower-left plots show the PL spectra, and the lower-right plots show the SIMS-derived In composition profiles. (d) HR-TEM image of an individual InGaN nanorod, with enlarged HAADF-STEM views of the boxed GaN region (green) and graded InGaN region (orange). (e) Atomic-resolution ABF-STEM image of the InGaN nanorod and a higher-magnification view of the cyan-boxed region.

Reversing the axial grading direction does not simply change the composition profile; it reverses the polarization-gradient-induced electrostatic profile. As illustrated in Figure 2a,b, the axial variation of polarization in the N-polar InGaN nanorods generates bound polarization charges within the graded region, with the charge sign determined by the In-composition gradient. Thus, for [0001̅]-oriented N-face crystals, grading from GaN to InGaN (In-increasing) is expected to produce negative bound charges, whereas reversing the gradient from InGaN to GaN (In-decreasing) produces positive bound charges. This charge reversal should generate opposite electrostatic profiles and correspondingly reversed near-surface band bending in the two nanorod arrays. To directly probe this buried electrostatic contrast, we performed AR-HAXPES on the N 1s core level. The depth sensitivity is controlled by the information depth,^43,44^ d ≈ 3λcosθ, which corresponds to the depth contributing 95% of the detected signal and decreases from ∼25 nm at 2° to ∼12 nm at 60°, where λ is the inelastic mean free path and θ is the emission angle measured from the surface normal. For N 1s photoelectrons excited at 6.5 keV, λ is estimated to be ∼8.2 nm from the TPP-2 M formula,^45^ and increasing θ therefore progressively enhances the near-surface sensitivity. As shown in Figure 2e,f, the broader N 1s line shape of the In-increasing nanorods indicates that photoemission contributions from different depths are distributed over a wider binding-energy range within the same escape depth, consistent with a stronger depth-dependent band-bending profile. To extract the underlying electrostatic profiles, we globally analyzed the full angular series, rather than relying only on the two limiting-angle spectra displayed in Figure 2e,f, using a depth-weighted photoemission model in which attenuation-weighted Voigt components from different depth layers were summed over the probing depth.^46,47^ This procedure constrains a single depth-dependent electrostatic profile for each sample; the intermediate-angle spectra at 33° and 48° are shown in Supporting Information Figure S3. The fitted depth components directly reveal the electrostatic reversal. In the In-increasing nanorods, the near-surface 1 nm component lies at higher binding energy than the deeper bulk-like component, consistent with downward band bending toward the surface. In contrast, the In-decreasing nanorods show the opposite ordering, with the 1 nm surface component shifted to lower binding energy, indicating upward band bending. This opposite surface-to-bulk energy ordering is resolved at both emission angles, while the 60° spectra further accentuate the near-surface contribution. The near-surface valence-band-maximum (VBM) profile was then reconstructed by combining the N 1s-derived electrostatic potential with the composition-dependent local valence-band edge, and the local conduction-band minimum (CBM) was obtained using the composition-dependent band gap. All energies are referenced to the Fermi level (E_F_ = 0); details of the fitting and reconstruction procedures are provided in Supporting Information Section S2. The resulting profiles yield near-surface band-bending values of V_BB_ = −0.51 eV for the In-increasing nanorods and V_BB_ = +0.56 eV for the In-decreasing nanorods, confirming that reversing the polarization gradient reverses the sign of the unloaded electrostatic response.

**Figure 2:**
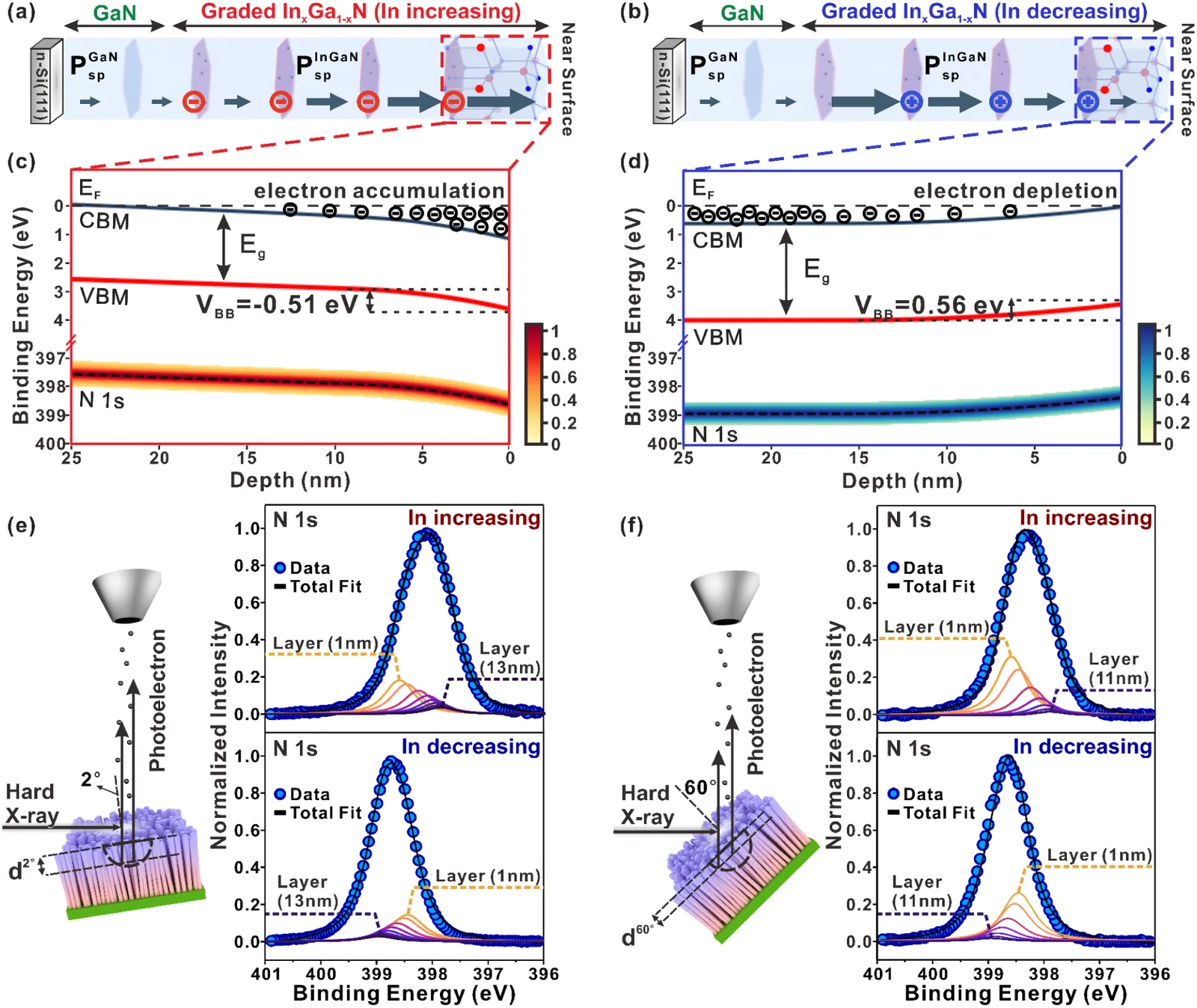
HAXPES-resolved reconstruction of the near-surface band structure in oppositely graded N-polar InGaN nanorods. (a, b) Schematic illustrations of the In-increasing and In-decreasing N-polar InGaN nanorods, respectively. The arrows denote the spontaneous polarization (*P*_sp_) in GaN and graded InGaN. Opposite grading directions generate opposite polarization-gradient induced bound charges, indicated by red and blue charge symbols. The red and blue dashed boxes indicate the near-surface regions reconstructed by HAXPES analysis; in the top surface region of the atomic models, the red and blue spheres denote metal and nitrogen atoms, respectively. (c, d) Reconstructed near-surface band diagrams from AR-HAXPES analysis for the In-increasing (c) and In-decreasing (d) nanorods. The color scale represents normalized N 1s intensity. (e, f) Left: schematics of the emission geometries used in the HAXPES measurements at 2° (e) and 60° (f). Right: HAXPES N 1s spectra of the In-increasing and In-decreasing nanorods acquired at 2° (e) and 60° (f). The colored curves show attenuation-weighted spectral components corresponding to different depths. At 2°, the spectral fwhm values are 1.095 and 0.935 eV for the In-increasing and In-decreasing nanorods, respectively; at 60°, the corresponding values are 1.038 and 0.914 eV.

This asymmetric band bending directly reflects grading-controlled carrier redistribution in unintentionally n-type InGaN nanorods: reversing the In-composition gradient reverses the polarization-bound-charge landscape, thereby driving mobile electrons toward or away from the nanorod top surface before mechanical actuation. In the In-increasing nanorods, the negative polarization-gradient-induced bound charges in the graded InGaN region drive mobile electrons toward the upper near-surface end of the nanorod, leading to electron accumulation and the downward band bending revealed by AR-HAXPES. Reversing the grading switches the polarization-gradient-induced bound charge from negative to positive, attracts electrons away from the surface and into the rod interior, and consequently produces near-surface depletion and upward band bending. These AR-HAXPES-derived unloaded electrostatic profiles define the starting point for nanogenerator operation. The experimentally resolved near-surface band-bending profiles, corresponding to electron accumulation and depletion near the nanorod tops, provide the electrostatic basis for the different carrier-screening behaviors during device operation, without requiring the two opposite grading directions to have identical local strain states in their buried GaN/InGaN interfacial regions.

To determine how this contrast manifests at the device level, we fabricated vertically integrated nanogenerators (VINGs) in a Cu/ITO/PMMA/InGaN/GaN/Si/Ag configuration (Figure 3a), in which PMMA fills the gaps between neighboring nanorods to enhance mechanical robustness, suppress interfacial leakage, preserve capacitive coupling to the top electrode, and limit direct internal screening.^1,48–51^ Under cyclic compression, strain-induced piezoelectric polarization P_pz_ is superimposed on the built-in electrostatic background established by the polarization-gradient-induced volume charge density ρ_G_ in the graded InGaN nanorods. The resulting piezoelectric charge modulation reshapes the band profile and generates a transient piezoelectric potential across the device (Figure 3c), which drives charge flow through the external circuit; upon release, this potential collapses and the current reverses, producing the alternating-current output schematically illustrated in Figure 3b. The output is therefore governed not only by the magnitude of the strain-induced polarization, but also by the unloaded carrier distribution established before compression, which determines whether the transient piezoelectric potential is rapidly screened or allowed to persist. Consistent with the HAXPES-resolved band profiles, the In-increasing VING delivers ∼3.1 V and ∼26 nA cm^–2^ under dark conditions, whereas the In-decreasing counterpart yields only ∼56 mV and ∼0.8 nA cm^–2^. As illustrated for the In-increasing device in Figure 3c, the unloaded electrostatic profile concentrates electrons near the surface, so that upon compression, the dominant screening remains largely confined to this region. Consequently, the deeper InGaN region contains fewer mobile electrons available to screen the strain-induced piezoelectric perturbation, allowing a larger piezoelectric potential to survive and be extracted through electrostatic coupling across the PMMA layer and the Si/Ag back contact. By contrast, in the In-decreasing nanorods, electrons remain distributed more broadly through the rod interior, enabling stronger dynamic screening of the strain-induced polarization throughout the active region and consequently much weaker output. Importantly, unlike conventional compositionally uniform piezoelectric nanorods, simply extending the graded region is not inherently beneficial in this system. The local grading-induced bound-charge density is given by *ρ*_G_(z) = −dP/dz; therefore, for a comparable overall polarization change ΔP, increasing the graded-region length Δz reduces its average magnitude, $\left|{\mathrm{\rho ̅}}_{G}\right|\approx \left|\Delta P/\Delta z\right|$ , and weakens the grading-induced redistribution of mobile carriers.

**Figure 3:**
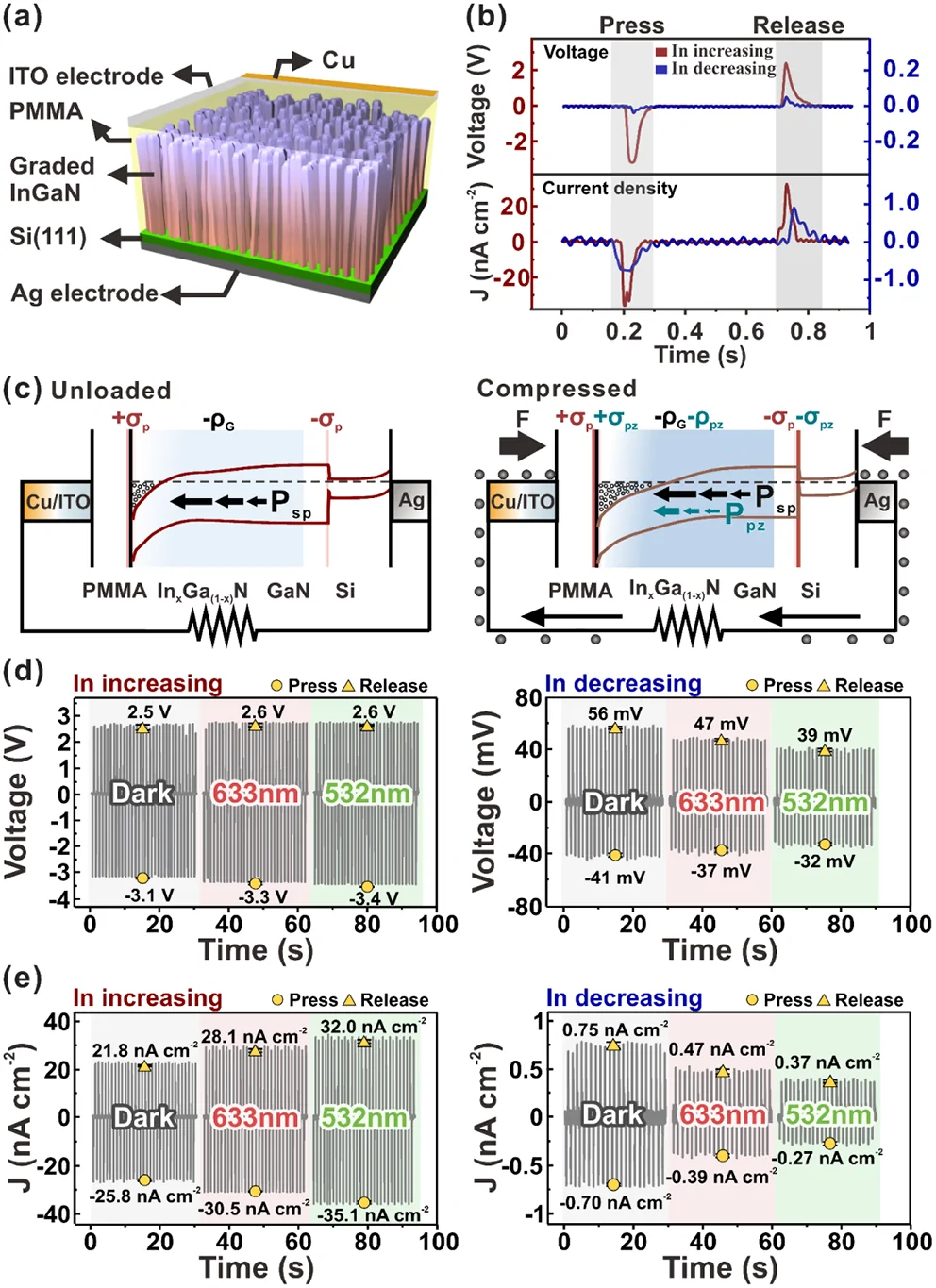
Vertically integrated InGaN nanogenerators: device design, band-structure-based operating mechanism, and grading-dependent photoresponse. (a) Schematic illustration of the VING incorporating InGaN nanorod arrays. (b) Single-cycle output voltage (upper) and current density (lower) signals of the In-increasing and In-decreasing VING devices. The shaded regions mark the press and release half-cycles. (c) Schematic energy band diagrams of the In-increasing VING device in the unloaded and compressed states. In the unloaded state, the electrostatic profile is established by polarization-gradient induced volume charge density (−*ρ_G_*) associated with the spatial variation of spontaneous polarization (P_sp_) in the graded InGaN nanorods, including interfacial sheet charge densities +σ*_p_* and −σ*_p_* arising from abrupt discontinuities in P_sp_ at the InGaN/PMMA and GaN/Si interfaces, respectively. Under compression, the strain-induced piezoelectric polarization (P_pz_) gives rise to additional interfacial sheet charge density (σ_pz_) and volume charge density (ρ_pz_) which are superimposed on the pre-existing polarization-gradient-induced charge distribution. In the In-increasing structure, these additional piezoelectric charges reinforce the pre-existing electrostatic asymmetry, thereby increasing the internal electrostatic potential difference and output voltage; the accompanying enhanced carrier redistribution then drives a larger transient charge flow through the external circuit. (d, e) Output voltage (d) and current-density (e) signals of the In-increasing (left) and In-decreasing (right) VING devices measured in the dark and under red (633 nm) and green (532 nm) illumination. Circles and triangles denote the cycle-averaged extrema in press and release states, respectively. Error bars indicate cycle-to-cycle standard deviations, typically on the order of ∼10^–2^ and 10^–3^ V in (d) and ∼10^–1^ and ∼10^–2^ nA cm^–2^ in (e), for the In-increasing and In-decreasing devices, respectively.

This grading-dependent electrostatic picture is further corroborated by the illumination-dependent device measurements in Figure 3d,e. Across all conditions, the In-increasing device sustains cycle-averaged peak voltages on the volt scale and cycle-averaged peak current densities in the tens of nA/cm^2^, whereas the In-decreasing device remains limited to tens of millivolts and sub-1 nA/cm^2^ output. Under illumination, this contrast becomes even stronger: in the In-increasing device, both voltage and current density increase progressively from dark to 633 nm (red) and 532 nm (green) excitation, whereas in the In-decreasing device, both outputs are progressively suppressed. Because 633 nm excitation lies below the dominant band-edge transition of the graded InGaN region, the red-light response may involve weak sub-band gap excitation through localized, band-tail, or defect-related states in the nanorods. These opposite photoresponses show that the grading-defined unloaded electrostatics determine whether photoexcited carriers assist external charge extraction or instead exacerbate the internal screening that suppresses useful piezoelectric potential during operation. Consistent with the conventional behavior of piezoelectric semiconductor nanogenerators,^1,7^ the In-decreasing device exhibits illumination-induced output reduction due to enhanced carrier screening of the piezoelectric potential. Conversely, in the In-increasing nanorods, photoexcited electrons are driven toward the preexisting near-surface accumulation region while holes are displaced in the opposite direction along the N-polar axis, confining dynamic screening near the surface and reducing compensation of the strain-induced piezoelectric potential in the deeper InGaN region. A larger internal potential is therefore preserved for extraction through the Si/Ag back-contact pathway. The hybrid response thus arises from the interplay of optical excitation with grading-defined carrier redistribution and internal electrostatics, making light and strain cooperative rather than competitive within the same nanorod device.

The grading-dependent operating mechanism is further clarified by exponential fitting of the press and release voltage transients (Figure 4a,b) and by rectification-and-storage demonstrations for hybrid energy harvesting, enabling LED lighting (Figure 4c). The fitted curves in Figure 4a yield characteristic decay times τ for the two half-cycles, and the key signature is their grading dependence (Figure 4b). In the In-increasing device, *τ* increases under illumination and becomes much longer under green than under red excitation, showing that photoexcitation does not accelerate screening of the remnant piezoelectric potential, but instead prolongs its survival by confining dynamic screening near the surface. Consistent with the angle-resolved HAXPES results, this surface-confined screening originates from the preexisting near-surface electron-accumulation profile, which reduces compensation of the strain-induced piezoelectric potential in the nanorod interior and allows a larger internal driving field to persist for longer durations. The stronger lifetime enhancement under green illumination further suggests that this effect becomes stronger as photocarrier generation in the graded InGaN increases. By contrast, the In-decreasing device shows no comparable enhancement: τ remains short and decreases slightly under illumination, indicating that the reversed electrostatic profile does not preserve a long-lived extraction-favorable charge configuration, but instead leaves the device screening-dominated. The capacitor-charging data in Figure 4c provide the time-integrated consequence of the same screening physics: because useful charge is delivered only while the rectified generator voltage exceeds the storage voltage plus the diode loss, the In-increasing device injects more charge into the 2.2 μF capacitor during each cycle, yielding a faster voltage rise and a higher saturation voltage, with charging performance following green > red > dark. The corresponding storage-capacitor charging model is provided in the Supporting Information Section S3. A red LED can be lit after charging ten 2.2 μF capacitors in parallel and reconnecting them in series, demonstrating hybrid charging capability rather than efficiency optimization. For broader performance context, a comparison with representative piezoelectric nanogenerators, hybrid mechanical–optical energy harvesters, and the present polarization-gradient-engineered nanogenerator is provided in Table S1 of Supporting Information Section S4.

**Figure 4:**
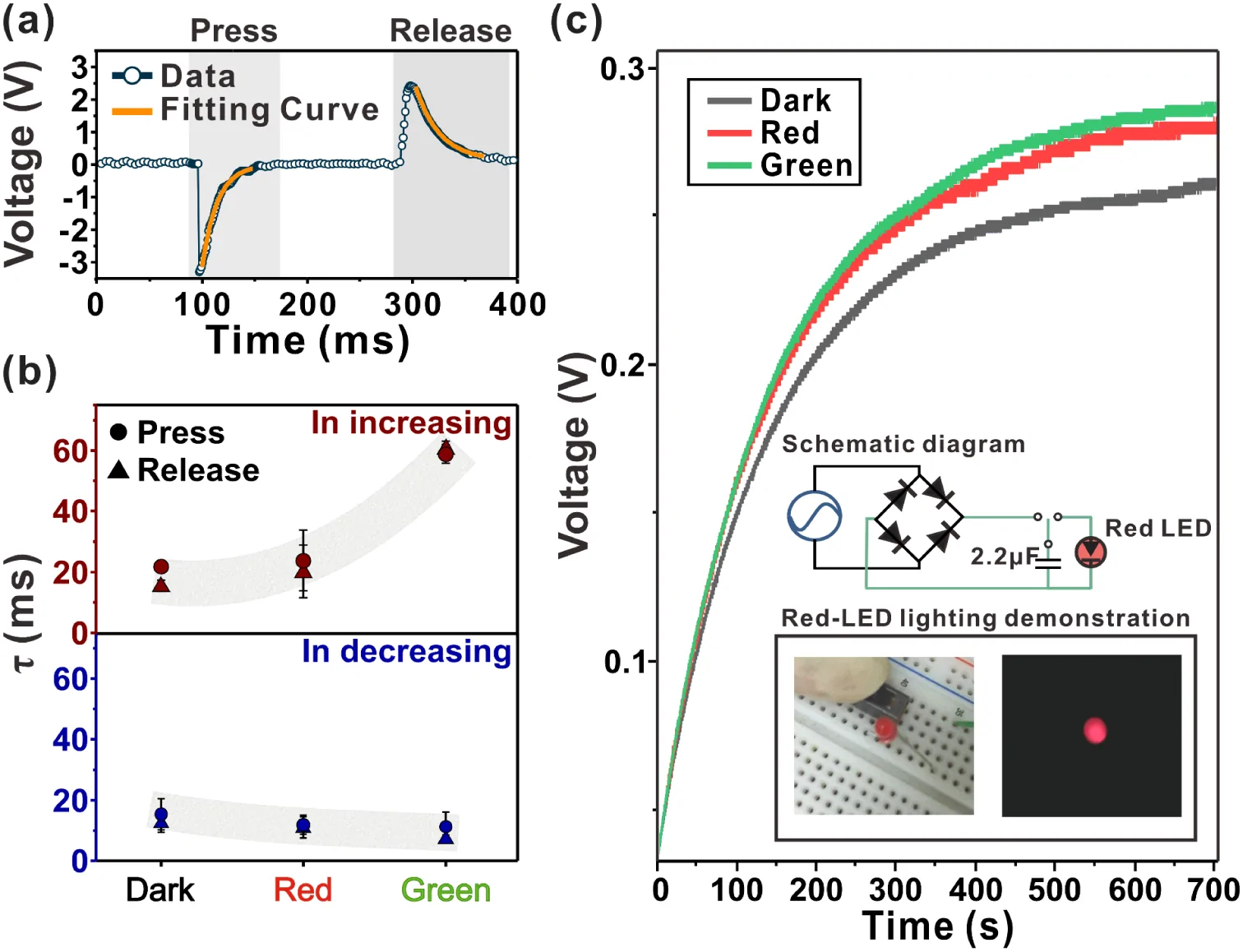
Transient decay characteristics and capacitor-charging performance of the InGaN nanogenerators. (a) Single-cycle transient voltage of the nanogenerator. (b) Decay time constants *τ* extracted from the press and release transients under dark, red-light, and green-light conditions. Error bars indicate cycle-to-cycle standard deviations of the extracted effective decay times of order 10^0^ ms for both devices. (c) Capacitor-charging curves obtained by rectifying the nanogenerator output to charge a 2.2 μF storage capacitor under dark, red-light, and green-light conditions. The circuit configuration used for rectification and energy storage is shown in the upper inset, and the lower inset shows the red-LED lighting demonstration driven by the harvested and stored energy.

## Conclusions

In summary, this work demonstrates that polarization-gradient engineering in N-polar InGaN/GaN nanorods provides a promising strategy for improving hybrid mechanical–optical energy harvesting in piezoelectric semiconductor devices. By combining depth-sensitive AR-HAXPES with nanogenerator measurements, we show that opposite grading directions generate opposite built-in electrostatic profiles, which in turn govern output level, photoresponse, dynamic behavior, and rectified energy-storage capability. The superior performance of the In-increasing nanorods highlights the importance of electrostatic design in preserving the strain-induced piezopotential and enabling more effective conversion of coupled ambient stimuli into usable electrical energy. More broadly, scalable and reproducible control of polarization-gradient profiles could extend this design principle to other compositionally graded polar-semiconductor nanoarchitectures for self-powered sensing and hybrid energy-harvesting systems.

## Methods

### Growth and Characterization of Compositionally Graded InGaN/GaN Nanorods

The Si(111) substrates were cleaned in dilute HF solution to remove the native oxide layer, rinsed in deionized (DI) water, and dried with nitrogen gas. Subsequently, the substrates were immediately loaded into a radio frequency plasma-assisted molecular beam epitaxy (PA-MBE) system (SVTA 35-Nitride MBE). Prior to growth, the substrates were thermally degassed at 800 °C for 1 h under an ultrahigh vacuum of ∼1 × 10^–9^ Torr, followed by cooling to the growth temperature of 730 °C.

Nitrogen plasma was ignited at a flow rate of 2 sccm and RF power of 500 W to establish nitrogen-rich conditions and the Ga effusion cell temperature was set to 900 °C. GaN nanorods were grown at 730 °C for 1 h. Subsequently, the substrate temperature was lowered to 530 and 540 °C to grow nanorods with increasing and decreasing In composition gradients, respectively. During the 8 h growth of the InGaN segments, the In K-cell temperature was gradually increased from 800 to 850 °C, or decreased from 840 to 790 °C, respectively, at a rate of approximately 0.1 °C per minute. This process was maintained under continuous nitrogen plasma (2 sccm, 500 W). After growth, all metal shutters and the nitrogen plasma were closed. The samples were cooled to room temperature at a rate of 5 °C per minute. The growth process was monitored in situ using reflection high-energy electron diffraction (RHEED) operated at 15 keV.

Scanning electron microscopy (SEM; Merlin, Zeiss) images were obtained at an accelerating voltage of 3 keV to characterize the plan-view and cross-sectional morphologies of the as-grown InGaN nanorods. The plan-view images were used to evaluate the nanorod areal density, while the cross-sectional images were used to assess their length and overall geometry. To investigate the optical emission properties, photoluminescence (PL) spectroscopy was performed at room temperature. A continuous-wave (CW) 325 nm helium–cadmium (He–Cd) laser (KIMMON) was used for excitation. The excitation beam was focused onto the sample through a 40× objective lens (NA = 0.49, Thorlabs). The PL signal was collected and directed to a Kymera 328i spectrometer (Andor) for spectral analysis. The depth-dependent indium profile was obtained by SIMS using a CAMECA IMS 7F secondary ion mass spectrometer equipped with a Cs primary ion source. The TEM and STEM characterizations were conducted on the aberration-corrected JEOL 2100F electron microscope, operated at 200 kV. The ABF and high-angle annular dark-field (HAADF) STEM are subject to the respective collection angles of 72–192 and 7.2–19.2 mrad, and the specimen was deposited on a Cu grid for the observations.

### Depth-Resolved Hard X-ray Photoelectron Spectroscopy (HAXPES)

Hard X-ray photoelectron spectroscopy (HAXPES) measurements were carried out at the Taiwan undulator beamline BL12XU of SPring-8 in Japan. The photon energy is set approximately 6.5 keV under an ultrahigh vacuum (UHV) environment with a base pressure of ∼1 × 10^–9^ Torr. The photoemission spectra were collected using an MB Scientific A-1 HE electron analyzer.

To obtain depth-resolved chemical states and band structure profiles of the InGaN nanorods, angle-resolved HAXPES measurements were conducted. The photoelectron emission angles were systematically varied at 2°, 33°, 48°, and 60° with respect to the surface normal. In this geometry, the measurement at 2° represents the bulk-sensitive condition (providing the maximum information depth), while increasing the emission angle to 60° significantly enhances the surface sensitivity. The overall energy resolution of the system was approximately 270 meV, and the binding energy scale of all HAXPES spectra was calibrated using the Fermi cutoff of a clean gold (Au) reference sample.

### Fabrication Process of Nanogenerator (NG)

The as-grown InGaN/Si sample was spin-coated with poly­(methyl methacrylate) (PMMA) and subsequently baked on a hot plate at 180 °C for 3 min to remove residual solvent and stabilize the polymer layer. A 150 nm-thick indium tin oxide (ITO) layer was then deposited onto the PMMA surface by electron-beam evaporation to serve as the top electrode. To facilitate electrical contact without obstructing the optical illumination area, a conductive copper foil was attached to the edge region of the ITO layer. For the bottom electrode, silver paste was applied to the backside of the Si(111) substrate. Finally, copper wires were connected to the copper foil and the bottom silver-paste electrode for device measurements.

### Nanogenerator Output Measurements under Mechanical Loading and Illumination

The output response of the vertically integrated nanogenerator (VING) was measured using a home-built mechanical loading system. A periodic compressive force of approximately 1 kgf (∼9.8 N) was applied over a device area of approximately 4 cm^2^ at a frequency of ∼1 Hz, corresponding to a nominal average compressive pressure of approximately 24.5 kPa. The output voltage and current were recorded using low-noise voltage and current preamplifiers, respectively (SR560 and SR570, Stanford Research Systems). For light-modulated measurements, an optical fiber was integrated into the loading setup to illuminate the device during operation. Continuous-wave 532 and 633 nm lasers were used separately, each with an incident power of 1 mW. Each beam was expanded to form an approximately 1 mm-diameter circular spot on the InGaN nanorod device, providing optical excitation over the active device area during mechanical cycling.

## Supplementary Material



## Notes

HAXPES geometry, depth weighting, attenuation-based modeling of angle-dependent N 1s and valence-band HAXPES spectra, reconstruction of near-surface VBM and CBM profiles, and storage-capacitor charging model (PDF)
