# Supplementary material for: Polarization-Gradient Engineering of InGaN Nanorods for Hybrid Mechanical–Optical Energy Harvesting
Source: ACS Nano. 2026 Aug 31;20(36):24893–901. doi: 10.1021/acsnano.6c09038 (PMC13585178; doi:10.1021/acsnano.6c09038)
Supplement: Supplementary file 1 [file nn6c09038_si_001.pdf]

Supporting Information

Polarization-Gradient Engineering of InGaN Nanorods for Hybrid Mechanical–Optical Energy Harvesting

Sheng-Shong Wong[1,2†], Zhen-You Lin[1†], Ya-Chi Chiu[1], Ming-Hao Lee[4,5], Ming-Wen Chu[4,5], Chia-Hao Chen[2*], and Chung-Lin Wu[1,2,3*]

[1]*Department of Physics, National Cheng Kung University, Tainan 70101, Taiwan*
[2]*National Synchrotron Radiation Research Center (NSRRC), Hsinchu 30076, Taiwan*
[3]*Center for Quantum Frontiers of Research and Technology (QFort), National Cheng Kung University, Tainan 70101, Taiwan*
[4]*The Key Consortium of Electron Microscopy, National Taiwan University, Taipei 10617, Taiwan*
[5]*Center for Condensed Matter Sciences and Center of Atomic Initiative for New Materials, National Taiwan University, Taipei 10617, Taiwan*

[†] These authors contributed equally to this work.

[*] Email: chchen@nsrrc.org.tw; clwuphys@mail.ncku.edu.tw

S1. TEM and atomic-resolution HAADF-STEM characterization of the In-decreasing InGaN nanorod

Low-magnification TEM and atomic-resolution HAADF-STEM analyses were performed to examine the structural quality and local lattice variation of the In-decreasing nanorod. No discernible extended defects were observed within the examined regions. The measured c-axis lattice periodicities were approximately 5.201 Å in the GaN region and 5.295 Å in the graded InGaN region, consistent with lattice expansion associated with In incorporation. Under the assumption of a fully relaxed lattice, the measured InGaN periodicity corresponds to an estimated In content of approximately 22%. These results are consistent with the structural characteristics observed for the In-increasing sample and enable a more direct comparison between the two grading directions.

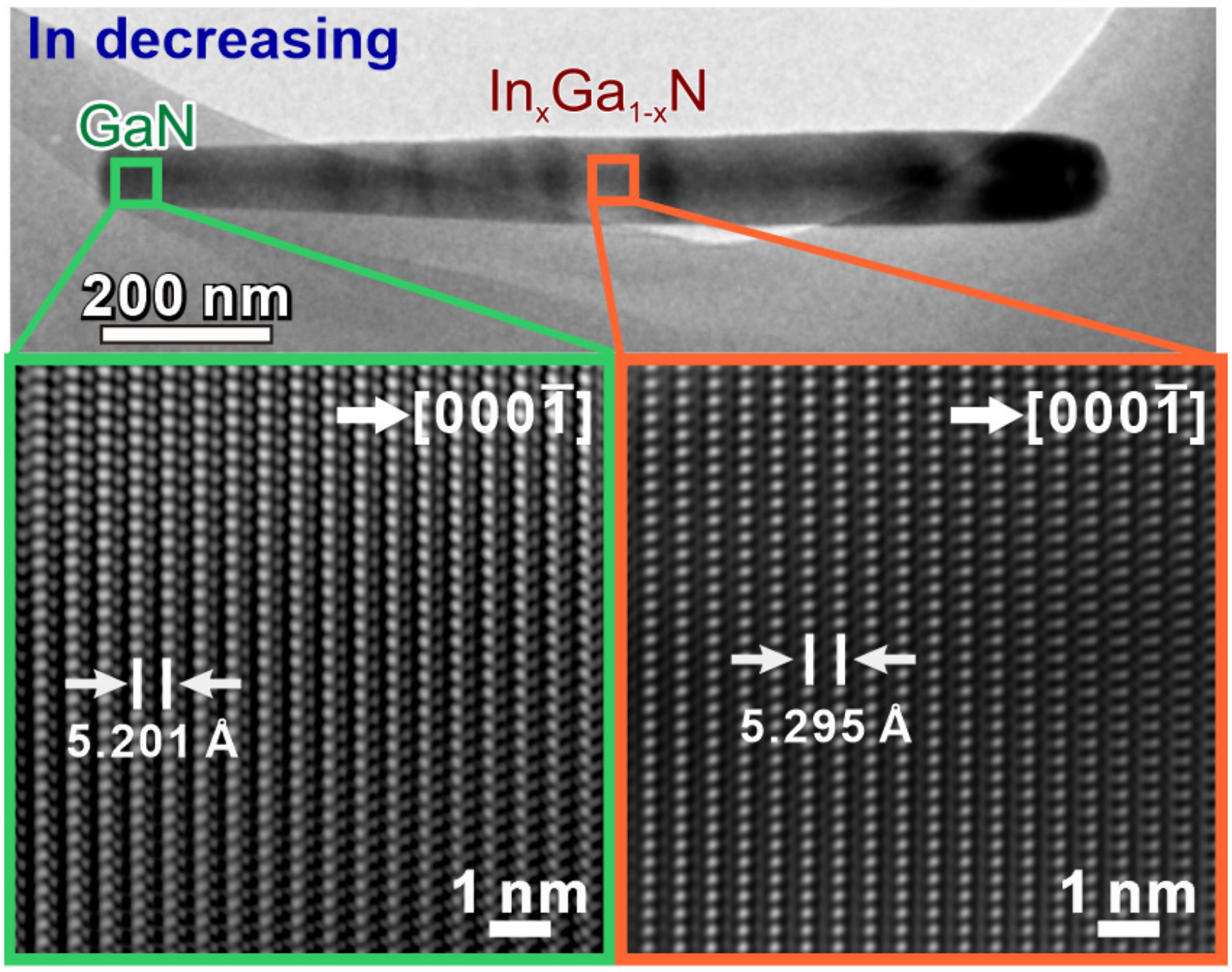

Figure S1 | Structural characterization of the In-decreasing nanorods. The examined nanorod exhibits high crystallinity without discernible extended defects in the imaged regions. Atomic-resolution HAADF-STEM measurements yield c-axis lattice periodicities of approximately 5.201 Å in the GaN region and 5.295 Å in the graded InGaN region, consistent with lattice expansion arising from In incorporation.

S2. HAXPES geometry, depth weighting, and spectral modeling

S2.1. Attenuation-based modeling of angle-dependent N 1s HAXPES spectra

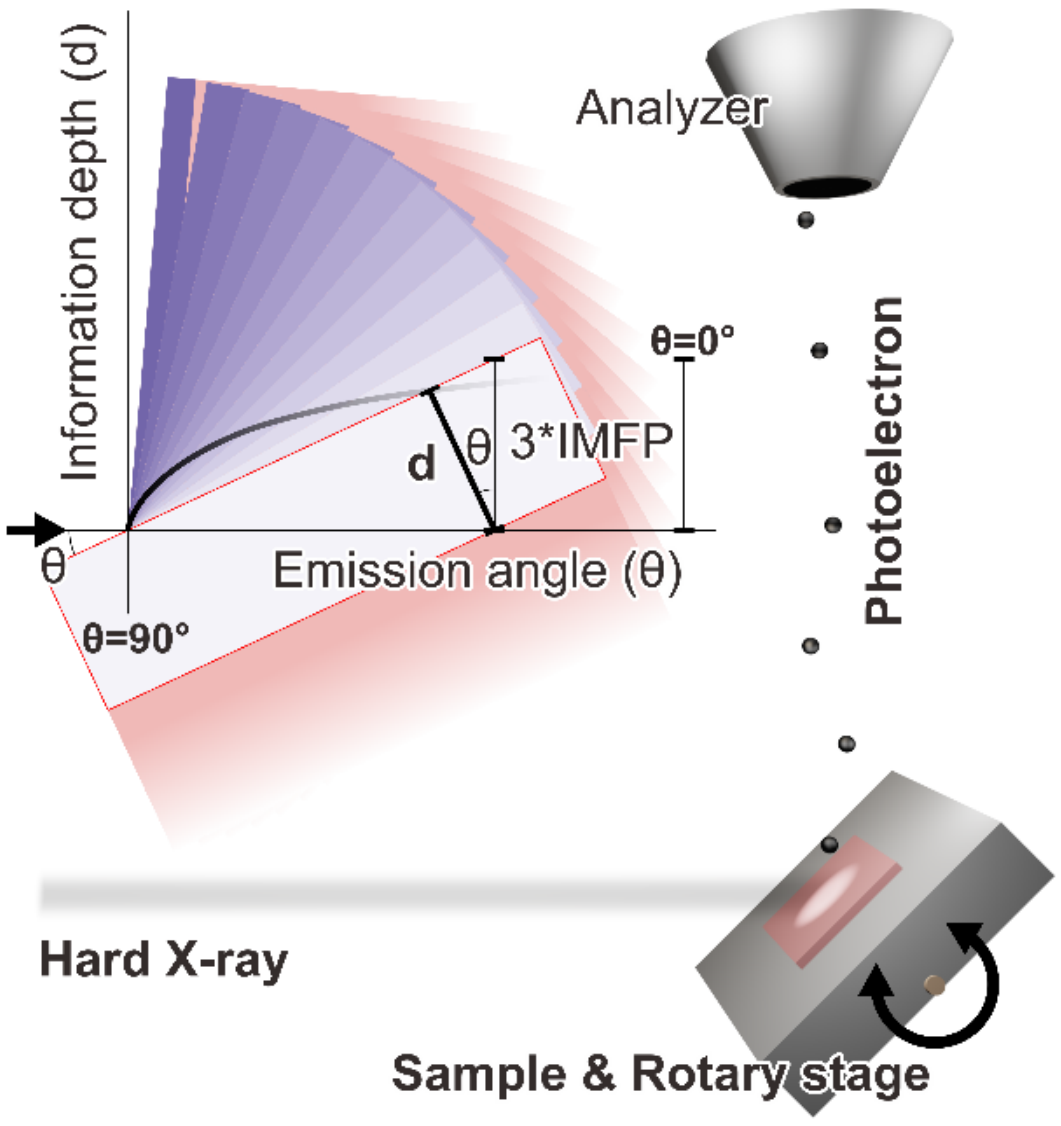

Figure S2 | Schematic of the AR-HAXPES geometry used to probe near-surface band bending.

Angular-resolved Hard X-ray Photoelectron Spectroscopy (AR-HAXPES) is a bulk-sensitive, non-destructive photoemission technique that enables electrostatic depth profiling. With the angle between the incident X-ray beam and the analyzer fixed, the sample was rotated to vary the photoelectron emission angle θ relative to the surface normal (Fig. S2). The inelastic mean free path (IMFP), λ, describes the average distance a photoelectron travels through the sample before undergoing inelastic scattering; for N 1s photoelectrons excited at 6.5 keV, λ is estimated to be ~8.2 nm using the TPP-2M formula. In angle-dependent measurements, the probing depth projected along the sample z direction is reduced by the geometric factor cosθ. The corresponding information depth (d), defined as the depth containing approximately 95% of the detected signal, can therefore be approximated as $d \approx 3\lambda\cos\theta$, corresponding to ~25 nm at 2° and ~12 nm at 60°. Increasing θ thus progressively enhances the near-surface sensitivity. The measured spectrum at each angle therefore represents an attenuation-weighted sum of photoemission contributions from different information depths. Because the depth-dependent shift of a core level reflects the underlying band-bending profile, the angular evolution of the N 1s spectra can be used to reconstruct the depth-dependent energy-shift profile.[1]

For quantitative analysis, the angle-dependent N 1s spectra were modeled using an attenuation-based spectral model.

$$\mathrm{I}(\theta, \mathrm{E}) \propto \int_0^{z_{max}} e^{-\frac{z}{\lambda cos\theta}} V(E - E_0(z); \sigma, \gamma)\, dz$$

, where $E_0(z)$ denotes the depth-dependent local N 1s center energy, and $V(E - E_0(z); \sigma, \gamma)$ is a Voigt function representing the local spectral contribution from a layer at depth z. The Lorentzian and Gaussian width parameters are given by

$$\gamma = \frac{FWHM_L}{2} \quad \sigma = \frac{FWHM_G}{2\sqrt{2ln2}}.$$

The Fermi cutoff of a clean Au reference sample was used to determine the experimental energy resolution; accordingly, the Gaussian broadening in the spectral simulation was fixed at $FWHM_G = 270\, meV$.

The depth-dependent binding energy, $E_0(z)$, was parameterized phenomenologically to represent the local electrostatic energy shift. The detailed profile of this shift depends on the local electrostatic environment near the top of the nanorod surface. As a simple approach, $E_0(z)$ was therefore described by a quadratic near-surface term extending from $z = 0$ to $z = z_l$, together with a residual linear term in the deeper region:

$$E_0(z) = \begin{cases} E_0^{Bulk} + m(z - z_l) + V_{BB}\left(1 - \frac{z}{z_l}\right)^2 & ,z < z_l \\ E_0^{Bulk} + m(z - z_l) & ,z \geq z_l \end{cases}$$

Here, E_0^{Bulk} denotes the bulk N 1s binding energy, V_{BB} is the amplitude of the quadratic near-surface band-bending contribution, z_l is its characteristic extension, and m describes an effective residual linear trend in the deeper region. This approach allows the characteristic extension, near-surface curvature, and deeper-region trend of the depth-dependent N 1s binding energy shift to be evaluated from the angle-dependent spectra. The global parameters ($E_0^{Bulk}, \gamma, \mathrm{m}, V_{BB}, z_l$) were optimized by simultaneously minimizing the least-squares error between the simulated and experimental N 1s spectra at all measured emission angles (Fig. S3). Because the measured peak position and linewidth represent a depth-weighted convolution of photoemission contributions in the presence of band bending, the angular evolution of the N 1s

spectra was analyzed using the full spectral model rather than by direct interpretation of the peak maxima.[2, 3]

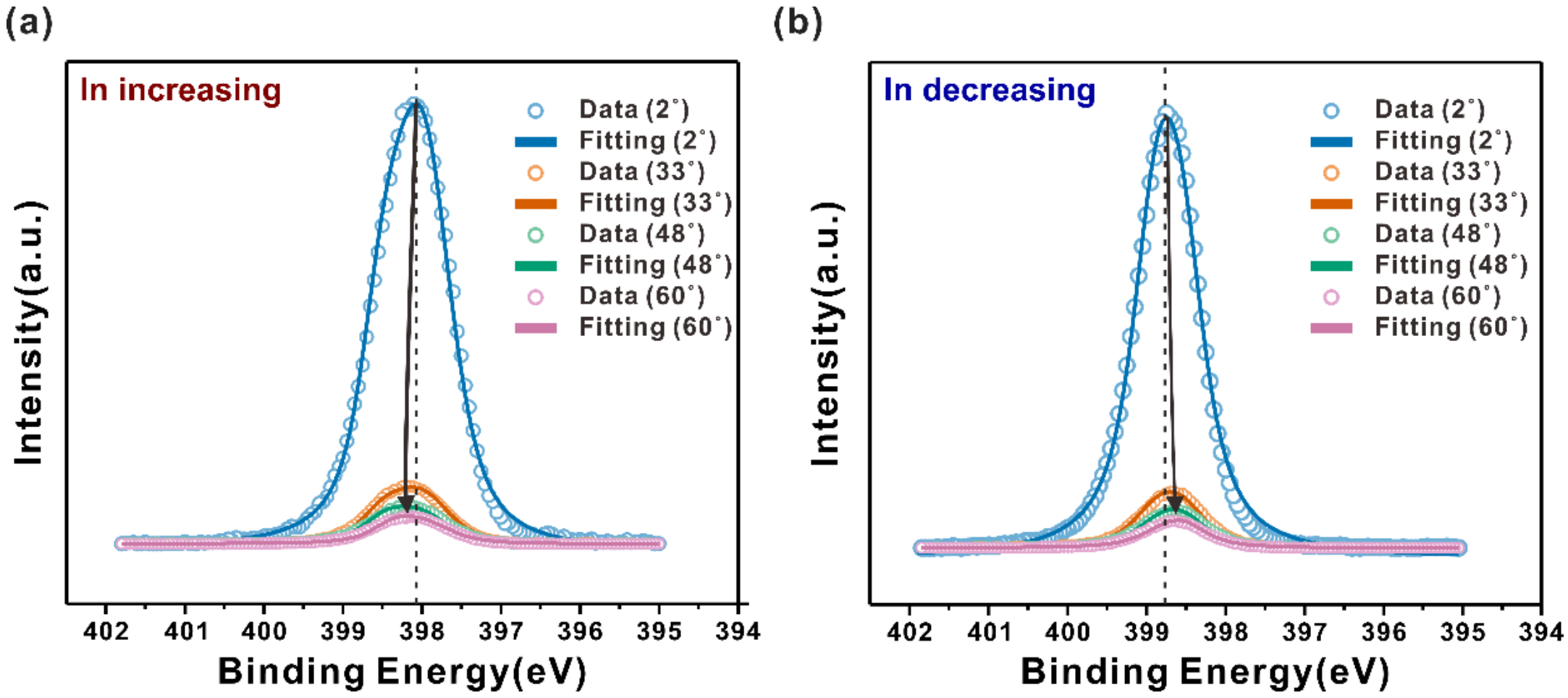

Figure S3 | Angle-dependent N 1s spectra for the (a) In-increasing and (b) In-decreasing samples. Open symbols and solid lines denote the experimental data and the corresponding calculated spectra, $\mathrm{I}(\theta, \mathrm{E})$, respectively. The vertical solid and dashed lines indicate the energy shifts.

S2.2. Reconstruction of near-surface VBM and CBM profiles

Because the local electrostatic potential shifts the core level and the band edges by the same amount, the N 1s-derived binding energy profile was used to reconstruct the near-surface valence-band-maximum (VBM) profile.[4] The local VBM binding energy profile was therefore obtained as

$$E_{VBM}^{BE}(\mathrm{z}) = E_{\mathrm{N1s}}^{BE}(\mathrm{z}) - \Delta_{\mathrm{N1s-VBM}}(\mathrm{x}(\mathrm{z}))$$

where $E_{\mathrm{N1s}}^{BE}(\mathrm{z})$ is the depth-dependent N 1s binding energy profile obtained from the AR-HAXPES analysis and $\Delta_{\mathrm{N1s-VBM}}(\mathrm{x}(\mathrm{z}))$ is the local In-content-dependent N 1s-to-VBM binding energy difference. At each depth, the local In composition $\mathrm{x}(\mathrm{z})$ was used to calculate the corresponding N 1s-to-VBM binding energy difference by interpolation between the reported GaN and InN reference values:[5, 6]

$$\Delta_{\mathrm{N1s-VBM}}(\mathrm{x}(\mathrm{z})) = (1 - \mathrm{x}(\mathrm{z}))\Delta_{GaN} + \mathrm{x}(\mathrm{z})\Delta_{\mathrm{InN}}$$

where Δ_{GaN} and Δ_{InN} are the reported N 1s-to-VBM binding energy differences for GaN and InN, respectively. The SIMS-derived $x(z)$ profiles were used directly in this reconstruction,

without imposing a linear composition-gradient assumption. Thus, the reconstructed VBM profile includes both the N 1s-derived electrostatic band bending and the composition-dependent N 1s-to-VBM offset.

The corresponding conduction-band-minimum (CBM) profile was then obtained from the reconstructed VBM profile using the local composition-dependent InGaN band gap:

$$E_{CBM}^{BE}(\mathrm{z}) = E_{\mathrm{VBM}}^{BE}(\mathrm{z}) - \mathrm{E_g}(\mathrm{x}(\mathrm{z}))$$

where $\mathrm{E_g}(x(z))$ was calculated from the GaN and InN band gaps with Vegard's law.[7] The resulting N 1s, VBM, and CBM depth profiles are summarized in Fig. S4.

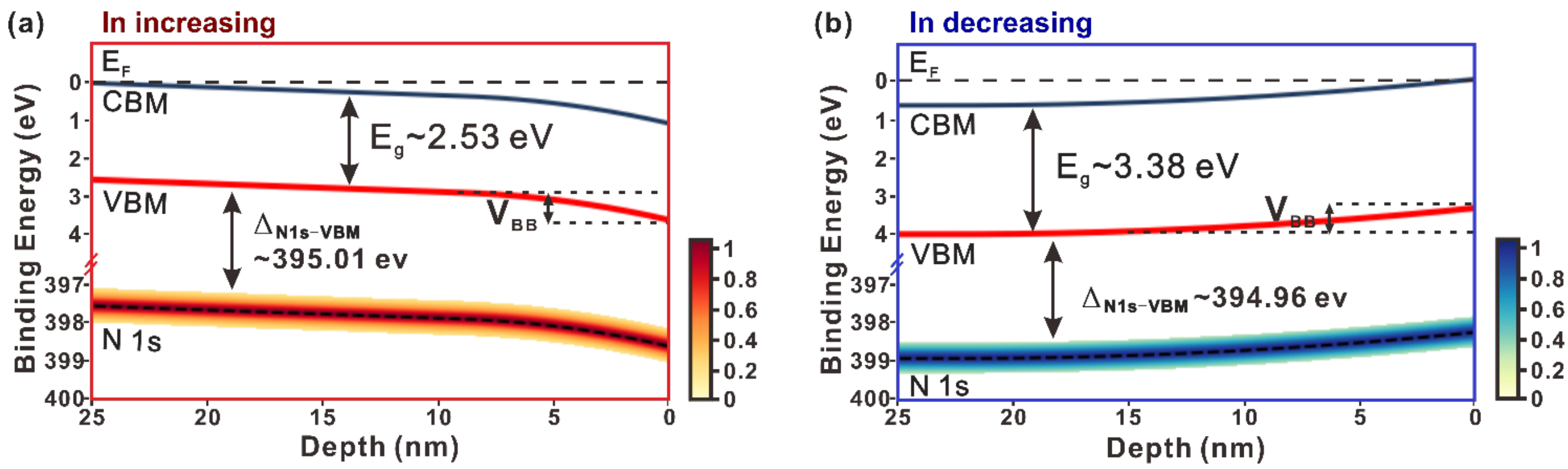

Figure S4 | Reconstructed near-surface band diagrams for (a) the In-increasing and (b) the In-decreasing samples. The colored maps show the normalized N 1s intensity distribution in each panel.

Furthermore, the angle-dependent valence-band spectra were used to verify the inferred band-bending direction, as shown in Fig. S5. The linearly extrapolated VBM positions, obtained from the valence-band leading edge and fitted background, exhibit opposite energy shifts for the two grading directions, in agreement with the N 1s core-level analysis. With increasing probing depth, the VBM shifts toward lower binding energy for the In-increasing sample and toward higher binding energy for the In-decreasing sample, corresponding to near-surface electron accumulation and depletion, respectively. Because these spectra contain attenuation-weighted contributions from multiple depths, they were used to evaluate the angular trend rather than to reconstruct a local VBM depth profile. The depth-dependent VBM profile was obtained from the

attenuation-based reconstruction of the N 1s spectra using the local In-content-dependent N 1s-to-VBM binding energy difference, $\Delta_{N1s-VBM}(x(z))$.

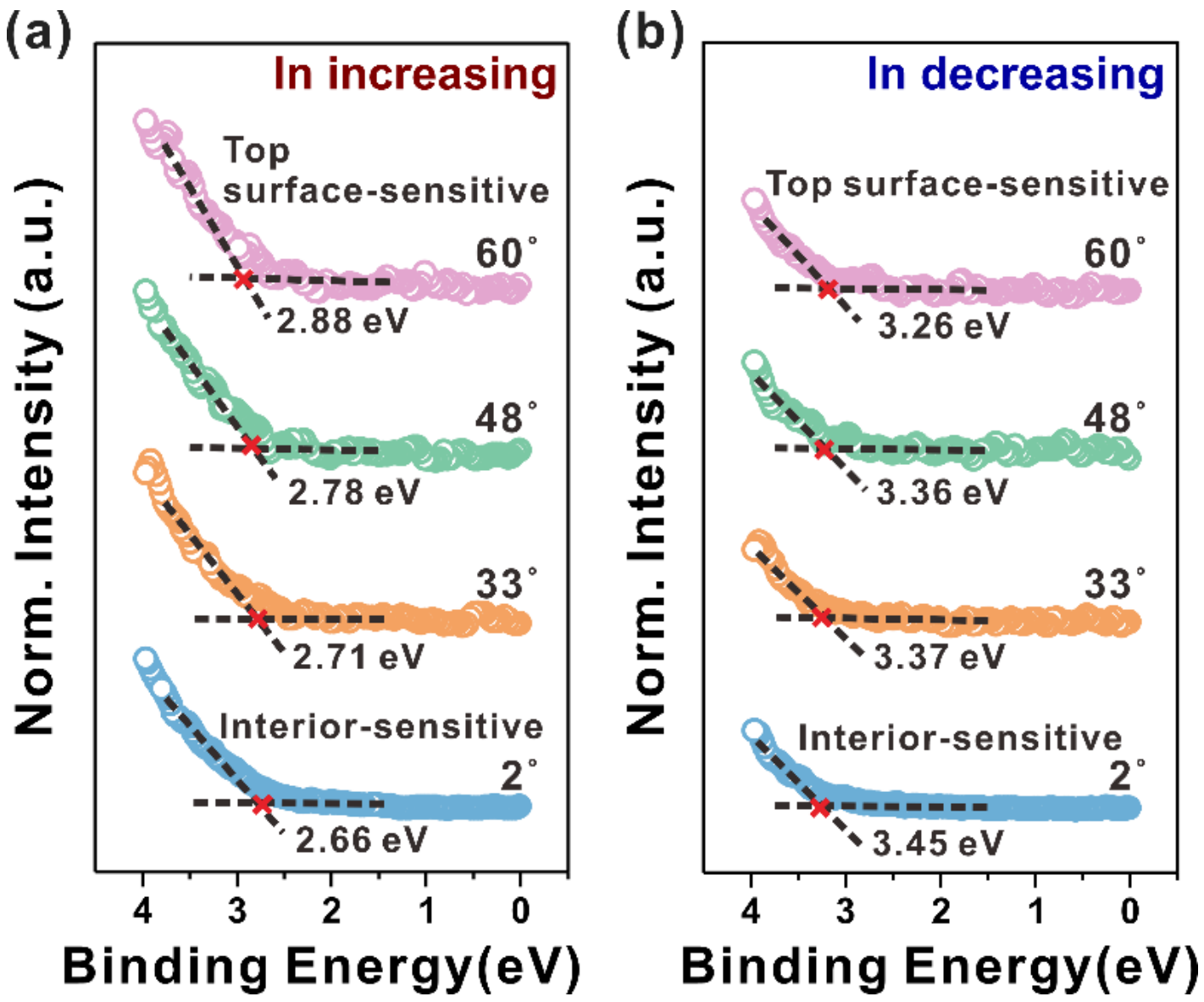

Figure S5 | Normalized angle-dependent valence-band spectra for (a) the In-increasing and (b) the In-decreasing samples. Red crosses mark the VBM positions determined by linear extrapolation of the valence-band leading edge to the fitted background. Open symbols denote the experimental data, and dashed lines denote the linear extrapolations of the valence-band leading edge and background.

S3. Storage-capacitor charging model

To relate the transient decay behavior to the storage-capacitor charging dynamics, each rectified charge-delivery pulse within one mechanical cycle was modeled using the same single-exponential form employed for the decay-time analysis.[8] Here, j indexes the individual charge-delivery events that contribute to charging during a cycle, typically corresponding to the two mechanical half-cycles. The transient voltage and current associated with the j-th charge-delivery pulse is represented as

$$V_j(t) = V_{0,j}e^{\frac{-t}{\tau_j}}, \qquad I_j(t) = I_{0,j}e^{\frac{-t}{\tau_j}}$$

where $V_{0,j}$ and $I_{0,j}$ are the voltage and current amplitudes for the j-th event and τ_j is the corresponding decay time. In this context, τ_j represents the dynamical response of the coupled device–circuit system, incorporating internal piezopotential screening and charge transfer through the device interfaces and external circuit.

Under rectified charging conditions, charge can be transferred to the storage capacitor only when the rectified generator-side voltage exceeds the sum of the capacitor voltage and the charging threshold.[9, 10] This condition can be expressed as

$$\left|V_j(t)\right| \geq V_c + V_{th}$$

where V_c is the instantaneous voltage across the storage capacitor and V_{th} represents the effective voltage threshold required for net charge transfer through the rectified charging path. This threshold is primarily associated with the rectifier turn-on loss. The corresponding effective conduction time for the j-th event is therefore

$$t_{eff,j} = \begin{cases} \tau_j ln(\frac{V_{0,j}}{V_c + V_{th}}) & , V_{0,j} > V_c + V_{th} \\ 0 & , V_{0,j} \leq V_c + V_{th} \end{cases}$$

The effective charge delivered to the storage capacitor during that event is then obtained by integrating the transient current over the above-threshold interval, giving

$$Q_j(V_c) = \int_0^{t_{eff,j}} I_j(t)\, dt = \begin{cases} I_{0,j}\tau_j(1 - \frac{V_c + V_{th}}{V_{0,j}}) & , V_{0,j} > V_c + V_{th} \\ 0 & , V_{0,j} \leq V_c + V_{th} \end{cases}$$

This expression shows that charge transferred in each event depends not only on the transient amplitude, but also on how long the rectified output remains above the effective charging threshold. Accordingly, for comparable amplitudes, a larger τ_j allows the transient to persist above threshold over a broader charging window and thus increases the charge that can be delivered during each event.

If the mechanical driving frequency is f, the cycle-averaged net charging current can be written as[11]

$$I_{net}(V_c) = f \sum_j Q_j(V_c) - \frac{V_c}{R_{leak}}$$

where R_{leak} is the effective leakage resistance. The evolution of the storage-capacitor voltage is then governed by

$$\frac{dV_c}{dt} = \frac{I_{net}(V_c)}{C_s} = \frac{f\ \sum_j I_{0,j}\tau_j(1 - \frac{V_c + V_{th}}{V_{0,j}}) - \frac{V_c}{R_{leak}}}{C_s} \text{ for } V_{0,j} > V_c + V_{th}$$

where C_s is the storage capacitance and the active charge-delivery events satisfying $V_{0,j} > V_c + V_{th}$. The charging rate is determined by the balance between the cycle-averaged rectified charge-delivery current and the leakage loss. Saturation is reached when

$$I_{net}(V_c) = 0$$

A longer transient voltage-decay time corresponds to a longer above-threshold charge-delivery window. When the transient amplitude is sufficiently large to exceed the charging threshold, a longer voltage-decay time extends the above-threshold charge-delivery window, allowing more charge to be transferred during each event and thereby increasing the cycle-averaged charging current. This picture is consistent with the experimentally observed charging behavior of the In-increasing device, for which green illumination produces the fastest capacitor-charging response and the highest final capacitor voltage.

S4. Comparison of representative piezoelectric nanogenerators, integrated mechanical–optical energy harvesters, and the present single-structure coupled nanogenerator

Table S1 compares the present polarization-graded InGaN/GaN nanorod device with three categories of previously reported systems: (i) representative ZnO-, $BaTiO_3$-, and $Pb(Zr,Ti)O_3$-based piezoelectric nanogenerators (PENGs), which provide established benchmarks for mechanical-energy harvesting; (ii) hybrid mechanical–optical harvesters, in which distinguishable photovoltaic (PV) or dye-sensitized solar-cell (DSSC) components are integrated with piezoelectric or triboelectric nanogenerator (TENG) units; and (iii) single-structure multi-effect coupled nanogenerators based on insulating ferroelectric oxides, in which optical energy is converted through the bulk photovoltaic effect within the same element.

Because the reported outputs depend strongly on active area, mechanical loading mode and magnitude, operating frequency, electrical load, illumination conditions, device configuration, and normalization method, the values in Table S1 are reproduced as originally reported. They are intended to provide a general comparison of representative performance and, in particular, of integration architecture, rather than a direct ranking of energy-conversion efficiency.

In the hybrid harvesters listed here, the optical and mechanical contributions originate from separate conversion pathways and combine additively, even when the components share a common material or are integrated into a single textile or stacked structure. In the single-structure

ferroelectric coupled nanogenerators, optical and mechanical conversion coexist in one insulating oxide element as parallel output channels; the photoresponse is fixed by the material, and the wide band gap (e.g., ~3.3 eV for $BaTiO_3$) limits visible-light utilization. By contrast, the present vertically integrated nanogenerator (VING) employs a direct-band-gap piezoelectric semiconductor in which the same polarization-graded InGaN nanorod region provides the piezoelectric conversion medium and defines the electrostatic environment governing illumination-dependent carrier redistribution. Visible light therefore does not contribute a separate photovoltaic channel but cooperatively enhances the piezoelectric output itself, with the sign of the photoresponse determined by the grading direction, and with visible absorption tunable through the alloy composition.

Table S1 | Comparison of representative piezoelectric nanogenerators, integrated mechanical–optical energy harvesters, and the present single-structure coupled nanogenerator

Category	Material & architecture	Energy inputs	Representative reported output	Optical–mechanical integration	Ref.
**Conventional PENG**	Lateral ZnO nanowire array on a flexible substrate	Mechanical bending	2.03 V, 107 nA; ~11 mW cm^{-3}	None (mechanical only)	*Nano Lett.* **10**, 3151 (2010)[11]
**Conventional PENG**	$BaTiO_3$ thin-film ribbons on plastic	Mechanical bending	1.0 V, 26 nA (0.19 µA cm^{-2}); ~7 mW cm^{-3}	None (mechanical only)	*Nano Lett.* **10**, 4939 (2010)[12]
**Conventional PENG**	Flexible PZT thin film	Mechanical bending	~200 V, 150 µA cm^{-2}	None (mechanical only)	*Adv. Mater.* **26**, 2514 (2014)[13]
**Hybrid harvester**	ZnO-NW DSSC stacked with ZnO-NW PENG	Solar + ultrasonic	~9 mV added by ultrasonic excitation	Separate DSSC subunit; outputs additive	*J. Am. Chem. Soc.* **131**, 5866 (2009)[14]
**Hybrid harvester**	Convoluted ZnO-NW solid-state hybrid cell	Solar + deformation	Combined output; not normalized for PENG comparison	Separate solid-state DSSC subunit; outputs additive	*Adv. Mater.* **23**, 873 (2011)[15]
**Hybrid harvester**	PVDF piezo/pyroelectric NG integrated with ZnO–P3HT heterojunction solar cell on flexible PET	Solar + mechanical (+ thermal)	Piezoelectric: 0.5 V, 20 nA; solar cell: 0.41 V, 31 µA cm^{-2} (AM 1.5); charged Li-ion battery to ~1.5 V	Separate PVDF NG and heterojunction PV subunits; outputs additive	*ACS Nano* **7**, 785 (2013)[16]

**Hybrid harvester**	Fiber solar cells woven with fiber TENGs (micro-cable textile)	Sunlight + motion	Charged 2 mF capacitor to 2 V in ~1 min	Separate PV and TENG fibers; outputs additive	*Nature Energy* **1**, 16138 (2016)[17]
**Hybrid harvester**	Self-healing TENG + PbS quantum-dot solar cell	Contact + solar	TENG: 24.6 V, 0.39 μA, 7.80 mW m^{-2}; PV: Jsc = 22.8 mA cm^{-2}, 4.92%	Separate TENG and QD-PV components; outputs additive	*Nano Energy* **125**, 109555 (2024)[18]
**Single-platform hybrid nanogenerator**	N-polar axially graded InGaN/GaN nanorod VING	Mechanical compression at ~9.8 N over 4 cm^2 at ~1 Hz + 633/532 nm (1 mW)	~3.1 V, ~26 nA cm^{-2} (dark); further enhanced under red/green illumination	No separate photovoltaic branch; Single graded InGaN structure; light modulates mechanically generated output.	This work

Note: Electrical outputs are listed as reported in the original publications. Direct quantitative comparison should be made cautiously because the active area, mechanical input, operating frequency, electrical load, illumination wavelength and intensity, device configuration, and output-normalization methods differ among the studies. The cited studies were selected to provide representative performance and architectural benchmarks rather than an exhaustive survey. PENG, piezoelectric nanogenerator; TENG, triboelectric nanogenerator; PV, photovoltaic; DSSC, dye-sensitized solar cell; QD, quantum dot; NW, nanowire; PVDF, poly(vinylidene fluoride); PZT, $Pb(Zr,Ti)O_3$; VING, vertically integrated nanogenerator; J_sc, short-circuit current density.

Reference

1. Lozac'h, M.; Ueda, S.; Liu, S.; Yoshikawa, H.; Sang, L.; Wang, X.; Shen, B.; Sakoda, K.; Kobayashi, K.; Sumiya, M. Determination of the Surface Band Bending in $In_xGa_{1-x}N$ Films by Hard X-Ray Photoemission Spectroscopy. *Sci. Technol. Adv. Mater.* **2013**, 14, 015007.
2. Wippler, D.; Wilks, R. G.; Pieters, B. E.; van Albada, S. J.; Gerlach, D.; Hüpkes, J.; Bär, M.; Rau, U. Pronounced Surface Band Bending of Thin-Film Silicon Revealed by Modeling Core Levels Probed with Hard X-rays. *ACS Appl. Mater. Interfaces* **2016**, 8, 17685–17693.
3. ViolBarbosa, C. E.; Shekhar, C.; Yan, B.; Ouardi, S.; Ikenaga, E.; Fecher, G. H.; Felser, C. Direct Observation of Band Bending in the Topological Insulator Bi_2Se_3. *Phys. Rev. B* **2013**, 88, 195128.
4. Xu, X.; Liu, X.; Guo, Y.; Wang, J.; Song, H.; Yang, S.; Wei, H.; Zhu, Q.; Wang, Z. Influence of Band Bending and Polarization on the Valence Band Offset Measured by X-ray Photoelectron Spectroscopy. J. *Appl. Phys.* **2010**, 107, 104510.
5. Duan, T. L.; Pan, J. S.; Ang, D. S. Interfacial Chemistry and Valence Band Offset between GaN and Al_2O_3 Studied by X-ray Photoelectron Spectroscopy. *Appl. Phys. Lett.* **2013**, 102, 201604.
6. Song, H. P.; Yang, A. L.; Wei, H. Y.; Guo, Y.; Zhang, B.; Zheng, G. L.; Yang, S. Y.; Liu, X. L.; Zhu, Q. S.; Wang, Z. G.; Yang, T. Y.; Wang, H. H. Determination of Wurtzite InN/Cubic In_2O_3 Heterojunction Band Offset by X-Ray Photoelectron Spectroscopy. *Appl. Phys. Lett.* **2009**, 94, 222114.
7. King, P. D. C.; Veal, T. D.; Jefferson, P. H.; McConville, C. F.; Lu, H.; Schaff, W. J. Variation of band bending at the surface of Mg-doped InGaN: Evidence of p-type Conductivity Across the Composition Range. *Phys. Rev. B* **2007**, 75, 115312.
8. Manrique, M.; Consonni, V.; Boubenia, S.; Roussel, H.; Zeghouane, M.; Labau, S.; Cavalaglio, S.; Pudda, C.; Jacob, V.; Le Rhun, G.; Salem, B. Enhancing the Output Voltage of Piezoelectric Nanogenerators Based on ZnO Nanowires Grown by Chemical Bath Deposition Using Compensatory Cu Doping. *Energy Technol.* **2024**, 12, 2301381.
9. Zi, Y.; Wang, J.; Wang, S.; Li, S.; Wen, Z.; Guo, H.; Wang, Z. L. Effective Energy Storage from a Triboelectric Nanogenerator. *Nat. Commun.* **2016**, 7, 10987.
10. Shu, Y. C.; Lien, I. C., Analysis of power output for piezoelectric energy harvesting systems. *Smart Mater. Struct.* **2006**, 15, 1499–1512.
11. Zhu, G.; Yang, R.; Wang, S.; Wang, Z. L. Flexible High-Output Nanogenerator Based on Lateral ZnO Nanowire Array. *Nano Lett.* **2010**, 10, 3151–3155.
12. Park, K.-I.; Xu, S.; Liu, Y.; Hwang, G.-T.; Kang, S.-J. L.; Wang, Z. L.; Lee, K. J. Piezoelectric $BaTiO_3$ thin Film Nanogenerator on Plastic Substrates. *Nano Lett.* **2010**, 10, 4939–4943.
13. Park, K.-I.; Son, J. H.; Hwang, G.-T.; Jeong, C. K.; Ryu, J.; Koo, M.; Choi, I.; Lee, S. H.; Byun, M.; Wang, Z. L.; Lee, K. J. Highly-Efficient, Flexible Piezoelectric PZT Thin Film Nanogenerator on Plastic Substrates. *Adv. Mater.* **2014**, 26, 2514–2520.
14. Xu, C.; Wang, X.; Wang, Z. L. Nanowire Structured Hybrid Cell for Concurrently Scavenging

Solar and Mechanical Energies. *J. Am. Chem. Soc.* **2009**, 131, 5866–5872.

15. Xu, C.; Wang, Z. L. Compact Hybrid Cell Based on a Convoluted Nanowire Structure for Harvesting Solar and Mechanical Energy. *Adv. Mater.* **2011**, 23, 873–877.
16. Yang, Y.; Zhang, H.; Zhu, G.; Lee, S.; Lin, Z.-H.; Wang, Z. L. Flexible Hybrid Energy Cell for Simultaneously Harvesting Thermal, Mechanical, and Solar Energies. *ACS Nano* **2013**, 7, 785–790.
17. Chen, J.; Huang, Y.; Zhang, N.; Zou, H.; Liu, R.; Tao, C.; Fan, X.; Wang, Z. L., Micro-Cable Structured Textile for Simultaneously Harvesting Solar and Mechanical Energy. *Nat. Energy* **2016**, 1, 16138.
18. Xiao, T.; Tu, S.; Tian, T.; Chen, W.; Cao, W.; Liang, S.; Guo, R.; Liu, L.; Li, Y.; Guan, T.; Liu, H.; Wang, K.; Schwartzkopf, M.; Fischer, R. A.; Roth, S. V.; Müller-Buschbaum, P. Autonomous Self-Healing Hybrid Energy Harvester Based on the Combination of Triboelectric Nanogenerator and Quantum Dot Solar Cell. *Nano Energy* **2024**, 125, 109555.
